# Developing a mobile health application for wound telemonitoring: a pilot study on abdominal surgeries post-discharge care

**DOI:** 10.1186/s12911-023-02199-z

**Published:** 2023-06-02

**Authors:** Tayebeh Baniasadi, Mehdi Hassaniazad, Sharareh Rostam Niakan Kalhori, Mehraban Shahi, Marjan Ghazisaeedi

**Affiliations:** 1 Department of Health Information Technology, Faculty of Para-Medicine, Hormozgan University of Medical Sciences, Bandar Abbas, Iran; 2 Infectious and Tropical Diseases Research Center, Hormozgan Health Institute, Hormozgan University of Medical Sciences, Bandar Abbas, Iran; 3 Department of Health Information Management, School of Allied Medical Sciences, Tehran University of Medical Sciences, Tehran, Iran; 4 Peter L. Reichertz Institute for Medical Informatics of TU Braunschweig and Hannover Medical School, Braunschweig, Germany

**Keywords:** Surgery, Wound, Surgical site infection, Postoperative period, Follow-up, Telehealth, mHealth

## Abstract

**Background:**

Many early signs of Surgical Site Infection (SSI) developed during the first thirty days after discharge remain inadequately recognized by patients. Hence, it is important to use interactive technologies for patient support in these times. It helps to diminish unnecessary exposure and in-person outpatient visits. Therefore, this study aims to develop a follow-up system for remote monitoring of SSIs in abdominal surgeries.

**Material and methods:**

This pilot study was carried out in two phases including development and pilot test of the system. First, the main requirements of the system were extracted through a literature review and exploration of the specific needs of abdominal surgery patients in the post-discharge period. Next extracted data was validated according to the agreement level of 30 clinical experts by the Delphi method. After confirming the conceptual model and the primary prototype, the system was designed. In the pilot test phase, the usability of the system was qualitatively and quantitatively evaluated by the participation of patients and clinicians.

**Results:**

The general architecture of the system consists of a mobile application as a patient portal and a web-based platform for patient remote monitoring and 30-day follow-up by the healthcare provider. Application has a wide range of functionalities including collecting surgery-related documents, and regular assessment of self-reported symptoms via systematic tele-visits based on predetermined indexes and wound images. The risk-based models embedded in the database included a minimum set with 13 rules derived from the incidence, frequency, and severity of SSI-related symptoms. Accordingly, alerts were generated and displayed via notifications and flagged items on clinicians’ dashboards. In the pilot test phase, out of five scheduled tele-visits, 11 (of 13) patients (85%), completed at least two visits. The nurse-centered support was very helpful in the recovery stage. Finally, the result of a pilot usability evaluation showed users’ satisfaction and willingness to use the system.

**Conclusion:**

Implementing a telemonitoring system is potentially feasible and acceptable. Applying this system as part of routine postoperative care management can provide positive effects and outcomes, especially in the era of coronavirus disease when more willingness to telecare service is considered.

**Supplementary Information:**

The online version contains supplementary material available at 10.1186/s12911-023-02199-z.

## Background

The first thirty days after surgery have been identified as a major focus area in patients’ care. The majority of complications are developed during this period, subsequently, early diagnosis and treatment of complications are critical [1, 2].

Surgical site infections (SSIs) are among the most common and serious complications following surgical procedures and the leading cause of surgical patients’ readmissions [3–5]. It accounts for nearly 20% of surgical readmissions [6]. Based on studies, the rate of SSIs in abdominal surgeries is higher than in other types of surgery [3, 7]. SSIs occur in 30–40% of patients undergoing abdominal surgery [8].

SSIs are associated with increased morbidity, mortality, extended patient recovery, treatment additional cost, and reduced patients' quality of life [9]. The financial burden of SSI is considerable; it ranks as the costliest hospital-acquired infection. It is estimated to be ranging from $3 billion to $10 billion annually in the US. Increased costs from SSIs are driven by the increased length of stay, readmissions, and emergency department visits [10].

The results of a systematic review to assess the prevalence of nosocomial infections (NIs) in Iran showed that SSI was one of the most common NIs observed. They emphasized that health policy-makers in Iran can help reduce NIs by improving the quality of the surveillance system [11]. Therefore, strategies for the surveillance of SSI are a national priority [9].

With increasingly shorter hospitalizations, many early signs of SSIs developed during the post-discharge period. Due to a lack of knowledge about particular SSI symptoms, these are inadequately recognized by patients [5]. Thus, it is important to develop and apply new solutions based on interactive technologies for patient support in these times. It helps to diminish unnecessary exposure and in-person outpatient visits [12].

Mobile health (mHealth) solutions with the development of smartphones as scalable, easy-to-use, as well as high-resolution photographic capability, have provided transmission of patient-generated health data and real-time communication [13–16]. A mobile app may be a useful technology for improving education, comprehension, and adherence to post-operative instructions [15]. Accordingly, it can facilitate early diagnosis of major surgery-derived complications such as surgical site infections (SSIs) and long-term monitoring of outpatient treatment [13, 14].

Some studies confirm the feasibility and safety of telemedicine-based services and mobile apps for the follow-up of discharged surgical patients. For example, Armstrong et al. (2017) showed that follow-up care could be delivered by a mobile app instead of in-person follow-up visits during the first 30 days after ambulatory breast reconstruction in post-mastectomy breast cancer [17]. In addition, another study reported the cost-effectiveness of this solution [18]. The result of Semple et al. (2015) study showed that at-home monitoring based on a mobile app for quality of recovery assessment was feasible and acceptable for surgeons and breast reconstruction and orthopedic patients [2]. In another study, Debono et al. (2016) indicated that mobile app home monitoring provided a useful tool for ambulatory spine surgery monitoring and minimized the need for in-person visits [12]. More specifically, Gunter et al. (2018), Sood et al. (2017), and Hwang’s (2016) showed that the smartphones-based apps for remote wound monitoring in different surgeries had clinical effectiveness, following their ability to earlier detect and then timely intervene on wound complications [5, 13, 19]. Generally, the new afore-mentioned follow-up system may affect positively the quality of postoperative recovery [20, 21].

Telemedicine use is emerging for surgical care and post-operative visits in low- and middle-income countries, and in developing countries, such as Iran [22]. In Iran, the implementation of this technology as a platform for post-acute care has been less addressed. It has received more attention in the management of chronic diseases such as heart disease [23] or diabetes self-care [24, 25].

Although in some countries patients are followed up after discharge, given its importance, this procedure is less common in Iran. In some centers, follow-ups are done after discharge by making telephone calls to patients. However, this method is not universal, and brings challenges (e.g. the lack of human resources or the unavailability of the patient) [26]. Accordingly, the overall follow-up rate of discharged surgical patients in Iran is low [26, 27]. Thus, the need to use telemedicine-based solutions is felt more. Hence, this study aims to develop a follow-up system for the remote monitoring of Iranian surgical patients. This study focuses on abdominal surgeries as a major group susceptible to complications and the detection of SSIs as major surgery complications.

## Methods

**Fig. 1:**
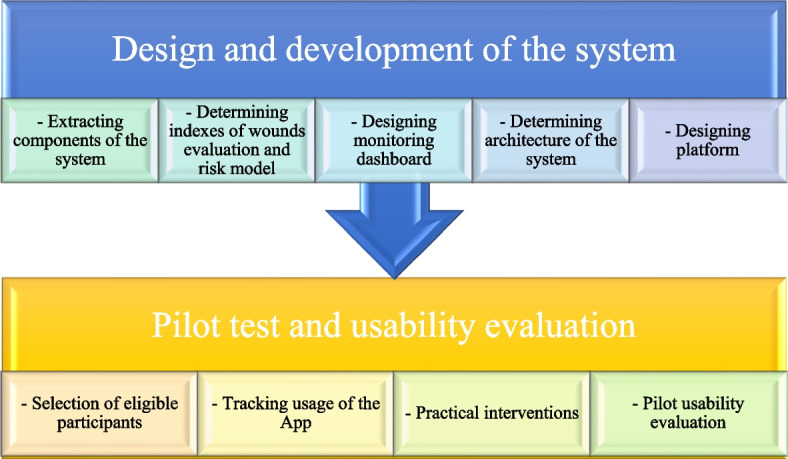
Phases of study

This applied study was performed in 2020 and conducted in the following two phases as represented in Fig. 1.

We reported our review in accordance with the Preferred Reporting Items for Systematic Reviews and Meta-analyses (PRISMA) guidelines [28].

### Design and development of the system

In the first stage of this study, a literature review was conducted. The searched main concepts consisted of a combination of “surgery”, “follow-up”, and “mHealth” terms. The search was carried out in databases of PubMed, Scopus, Embase, and Web of Science by review of English original articles between January 2009 and June 2019. Because of the possibility of limited coverage of studies related to mHealth-based follow-ups in abdominal surgeries, thus the search was carried out regardless of surgery type. Articles were included if they apply mHealth apps in a follow-up manner for surgery patients. Finally, based on the research question, qualitative data including the main features and components of follow-up systems were extracted from related articles [2, 5, 12, 13, 15, 17, 19, 29–45].

In addition, we sought to explore the specific needs, concerns, problems, or questions of abdominal surgery patients in the post-discharge period and to gather user-preferred content for a mobile-based follow-up system. Thus, we planned phone calls within one to two weeks after discharge. Accordingly, fifty patients discharged from the general surgery ward of a general academic hospital in the south of Iran participated in this phase.

Different classifications of data elements and features were obtained from the literature review and patients’ viewpoints stages. To enrich the extracted data and for their summarization and categorization in a scientific manner, we held a focus group discussion with the participation of the project team. They were from various professional disciplines. Based on the opinions of experts, after removing non-practical classification, initial modeling and main modules of the system were determined. In this meeting, information obtained from other resources such as hospital standard forms, patients' medical records, guidelines, theses, and reliable Iranian websites affiliated with the Ministry of Health were examined. In the next phase, a questionnaire along with a low-fidelity interface prototype (for a better understanding of the system) was designed for validating data elements and features by clinical experts (*n* = 30) through the Delphi technique. The questionnaire includes three sections (basic components, indexes for wound evaluation, and risk model). Additionally, at the end of each section, a free text box was considered for recording any new experts’ suggestions (Additional file 1). Items of the questionnaire were scored based on a five-option Likert scale (from 1 to 5); the scores of 5 and 1 belong to the most important and least important items, respectively. Thus, the components that were agreed upon by less than 50% (scores below 2.5 from 5) were removed. The components for which the collective agreement was more than 75% (scores of 3.75 to 5) remained as the main requirements. The agreement between 50 and 75% (scores of 2.5 to 3.75) resulted in a re-survey in the next Delphi round.

Experts for the Delphi phase were from Tehran, Iran, Shahid-Beheshti, and Hormozgan universities of medical sciences. These individuals were selected through purposive non-random sampling.

In this phase, the opinions of participants about the information structure of the prototype and its characteristics were gathered.

In the next phase, after modelling and confirming the basic architecture of the system, the database was designed in Microsoft SQL Server 2016 environment. Then for the development of the system, the design was done in Microsoft Visual Studio 2019 Preview using C#.Net programming language with.Net Core 2.2 framework. Encryption was used to send data to the database. The static and dynamic reporting was created using the above programming language queries. The templates in Gull were used to design the dashboards.

In this study, Progressive Web Applications (PWA) [46] capability was used to enhance user experience in desktop and mobile devices with different operating systems. Therefore, it is possible to have applications running on both Android and iOS devices in a faster manner.

### Pilot test and usability evaluation

For this phase, a pilot study was conducted in a large academic hospital in southern Iran (Shahid-Mohammadi hospital affiliated with Hormozgan University of Medical Sciences located in Bandar-Abbas city). Thus, this phase was performed in the general surgery ward of the hospital with the cooperation of abdominal surgery patients, interested volunteer doctors and nurses. At this stage, for volunteer patients, inclusion criteria were 1) open abdominal surgery inpatients, 2) at least being 18 years of age, 3) people who have a smartphone and mobile Internet, 4) ability to understand spoken and written Persian (Farsi), 5) literacy and ability to work with the app, and 6) willingness to use the mobile app after discharge. In addition, the exclusion criteria included 1) other types of surgery, 2) having visual and hearing impairments, and 3) mental disorders.

We evaluated the literacy and ability to work with the app, through patients’ self-declaration, checking the experience of working with similar apps, and a short test that was done before discharge to objectively determine the ability to work with the current app as well.

Nurse/physician practitioners were selected for this role because they were familiar with patients during their hospitalization stay and were determined to be best able for care continuity. In addition, they had higher information technology literacy than others and experience working with similar systems.

Eligible patients were identified on different days over two weeks. Then, adequate explanations and training were provided to patients regarding the purpose of the study and using the program. Informed consent was obtained for voluntary participation. At this time, necessary training and relevant instructions were also provided to the healthcare providers. After the preparation phase, patients were monitored and followed up through the platform for 30 days after discharge. In the pilot test, the way the app was used by the participants in the follow-up period was investigated. For this purpose, we tracked logging into the app, viewing educational materials, completing visits, sending messages and uploading wound images and other documents.

Furthermore, practical interventions were planned and implemented. In the meantime, a series of interventions have been done automatically after the data were registered by the patient. This was related to the patient's pre-discharge self-assessments. In addition, some of the interventions were carried out by clinical consultants after receiving the reported data from the remote visits. This included patients who had special problems based on the pre-defined risk-based models and reported symptoms. Note that a nurse and a doctor were assigned to be responsible for each patient, and the data could be seen by both. At the first level, nursing interventions and, if necessary, physician interventions were carried out. In addition, the patient could directly send messages to the doctor, nurse and even other consultants defined in the system through the chat service. Finally, the evaluation was done. For usability evaluation of the system, first, the initial version of the system was provided to inpatients (*n* = 5) as well as to the head nurses, general surgery, and infectious diseases specialists (*n* = 5). Suggestions for improving the system were received. After the collective agreement of the project team, these were applied in the final version. Finally, the usability of the system was quantitatively evaluated by the participation of patients and clinicians from Shahid-Mohammadi hospital that actively involved in the previous phase. For this purpose, a questionnaire derived from the mHealth App Usability Questionnaire (MAUQ) [47] was used (Additional file 2). After translating the questionnaire into the Persian language, its content and face validity were confirmed by nine experts (health information management (*n* = 3), medical informatics experts (*n* = 3), nurses(*n* = 2), and one general practitioner). After making minor changes to the questionnaire based on experts' opinions, the items were finally scored on a five-option Likert scale. Scores from 5 and 1 belong to the highest and lowest scores, respectively.

Generally, in the current study, User-Centered Design (UCD) guidelines with a repetitive approach for testing and refining requirements were applied. UCD methodology involves users throughout the design process. It uses iterative design cycles to increase the ultimate usability [48]. In addition, we considered Jakob Nielsen's 10 general principles for interaction design [49] to increase usability and improve the interface, and simplify data flow in the system.

### Ethics approval

This study was carried out in accordance with the research protocol and the ethical standards of the Helsinki Declaration and was approved by the Research Ethics Committee of Tehran University of Medical Sciences, Tehran, Iran (ethical code: IR.TUMS.SPH.REC.1397.194). The privacy of patients was protected by non-identifiable information. Also, consent was obtained from all participants before being involved in the study. During the research, voluntary participation, information confidentiality, and the right to withdraw at any time were considered. Generally, all methods were carried out under relevant guidelines, regulations, and intra-organizational ethical charters. This research was guaranteed not to change current standards of care.

## Results

The developed electronic follow-up system consists of a mobile health application (mHealth app) as a patient portal and a web-based platform for remote monitoring and patients' follow-up by the healthcare provider.

### System development

#### Components of the system

Based on the search strategy in the literature review phase, first, 368 articles were retrieved from four databases. Finally, 23 related articles were comprehensively surveyed for final analysis. Qualitative analysis of research-based apps showed that the common components of surgical patients' follow-up apps consist of tele-visits by examining of quality of recovery (QoR) or other indicators [2, 5, 12, 13, 15, 17, 29–32, 34–36, 38, 40–42, 44, 45], assessing post-operative body function and pain level status by virtual analog scale (VAS) [2, 12, 15, 17, 29, 32, 36, 40, 41, 44]; wound telemonitoring via recorded and transmitted SSI-related symptoms and images [2, 5, 13, 17, 19, 29, 35, 36, 40–43], teleconsultation, and patient connections with clinical staff [19, 30, 31, 38–40, 43], reminders and alerts [15, 33, 34, 40, 41, 45], and patient education [15, 33, 34, 39, 42, 43, 45]. In most studies, fourteen [5, 12, 19, 30, 31, 39, 42, 44] to thirty day [2, 17, 29, 34, 40, 41] follow-up period was planned for different surgeries.

**Fig. 2:**
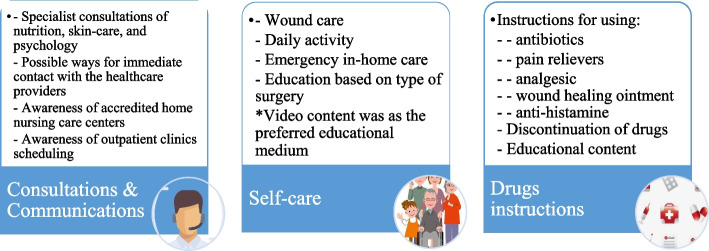
The extracted concepts and post-discharge needs from the patient's perspective

The extracted major themes from patients’ points of view related to post-discharge requirements are summarily presented in Fig. 2.

According to the agreement level of 30 experts (Table 1), the basic information infrastructure and the key function of the system were determined as represented in Table 2.

**Table 1:** Characteristics of the experts’ panel who took part in the Delphi phase

	**Group**	**Number (%)**	**Total**
**Gender**	Male	13(43.3)	30 (100%)
	Female	17(56.7)	
**Work experience**	< 5 Yrs	4(13.3)	16.2 ± 8.22 Yrs
	5–10 Yrs	5(16.7)	
	10–20 Yrs	9(30)	
	> 20 Yrs	12(40)	
**Physician** **(specialties & subspecialties)**	Cosmetic/Plastic Surgery Subspecialty	2(7)	3(10%)
	Vascular surgery Subspecialty	1(3)	
	General surgeons	9(30)	17(56.7%)
	Infectious diseases specialists	4(14)	
	Obstetricians and gynecologists	2(7)	
	Anesthesiologists	1(3)	
	Hospital chief	1(3)	
**Senior nurse**	Nurse manager	1(3)	7(23.3%)
	Matron	1(3)	
	Infection control supervisor	1(3)	
	Hospital accreditation supervisor	1(3)	
	Patient education supervisor	1(3)	
	Head nurses of general surgery wards	2(7)	
**Others**	Medical informatics expert	1(3)	3(10%)
	Health information management expert	2(7)

**Table 2:** The information infrastructure and key feature of the system based on the Delphi results

Mobile App components		Mean (SD^*^)	Acceptable	Web-based Platform components		Mean (SD^*^)	Acceptable
Patient Portal	Surgery-related documents	4.63 (0.60)	√	Care Team Portal	Discharge Instructions and clinical data management	4.67 (0.50)	√
	Self-management service	4.64 (0.70)	√		Educational content management	4.30 (0.97)	√
	Remote follow-up visits	4.40 (1.08)	√		Smart monitoring and surveillance dashboard	4.33 (1.01)	√
	Patient-provider communication service	4.50 (0.72)	√		Intervention management and telecommunication	4.30 (0.82)	√
	Patient-patient communication service and sharing experience	2.40 (0.70)	×		Educational panel for clinicians	4.30 (1.00)	√
					Static reporting	4.53 (0.01)	√

^*^SD Standard deviation

#### Indexes for surgery wounds evaluation

For the evaluation of surgery wounds, twenty indexes based on four criteria were validated and approved according to the experts’ agreement (Table 3).

**Table 3:** Accepted indexes for evaluation of surgery wounds

Criteria	Indexes	Mean (SD^*^)
Specific symptoms of wound infection	Increased redness Warmth Tenderness High swelling, bulging, stiffness, and inflammation Wound drainage (Green, White, Yellow) Foul smell from the wound Wound dehiscence High bleeding of wounds Sudden and exaggerated pain at the surgical site (use of analgesic)	4.61 (0.51)
General Symptoms of infection	Fever, chills, unusual sweating, dizziness, weakness, fatigue, lethargy, and pains	4.86 (0.35)
Overall satisfaction with wound healing process	Dissatisfaction, relative satisfaction, or complete satisfaction	4.18 (0.76)
Surgical wound images	Qualitative assessment of uploaded wound images	4.61 (0.72)

^*^SD Standard deviation

Scheduling for the tele-visits was set on times, 48 to 72 h after discharge as the first visit then post-discharge days 7, 14, 21, and 30 (overall 30-day follow-up). The emergency visits were available to receive information between the assigned times as well.

#### Risk model

Based on experts’ agreement level, risk-based models included a minimum set with 13 rules derived from incidence, frequency, and severity of SSI-related symptoms, which are embedded in the database (Table 4).

**Table 4:** Some IF–THEN rules

IF		THEN	
Symptom	Occurrence /Frequency	Alert type: Flag	Risk type
Fever/Chills	Yes/ [Most of the time or Always]	(Red)	Infection
Wound drainage (Green, White, Yellow)	Yes/ [Evident at the self-assessment time]	(Red)	SSI
High swelling, bulging, stiffness, and inflammation	Yes/ [Evident at the self-assessment time]	(Red)	SSI
Wound dehiscence	Yes/ [Evident at the self-assessment time]	(Red)	SSI
High bleeding of wounds	Yes/ [Evident at the self-assessment time]	(Red)	SSI
Wound drainage & Abdominal Pain	Yes &Yes / [Evident at the self-assessment time & Always]	(Red)	SSI

#### Monitoring dashboard

The information reported by patients on the dashboard screen is summarized and visible in a highlighted manner. Alerting clinicians via notifications and flagged items on smart monitoring dashboards for early detection of wound complications was considered.

#### General architecture of the system

**Fig. 3:**
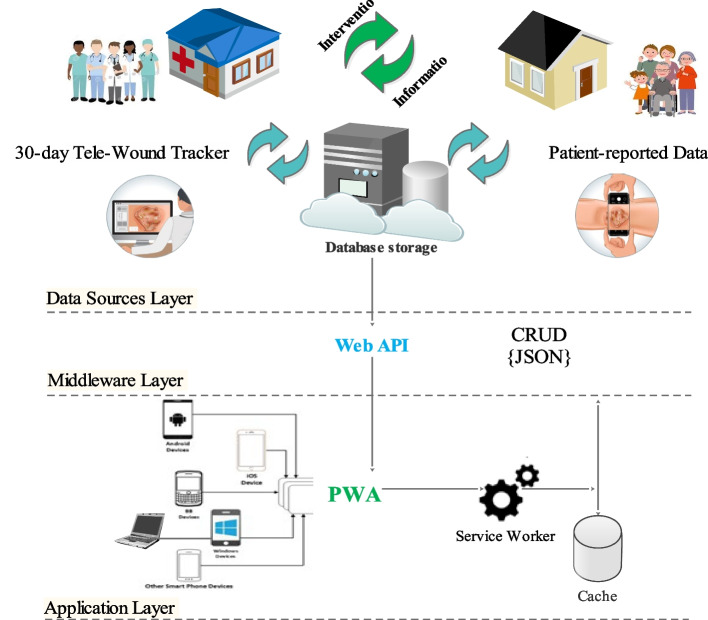
The general architecture of the wound telemonitoring system

The general architecture of the system is shown in Fig. 3.

#### Developed platform

**Fig. 4:**
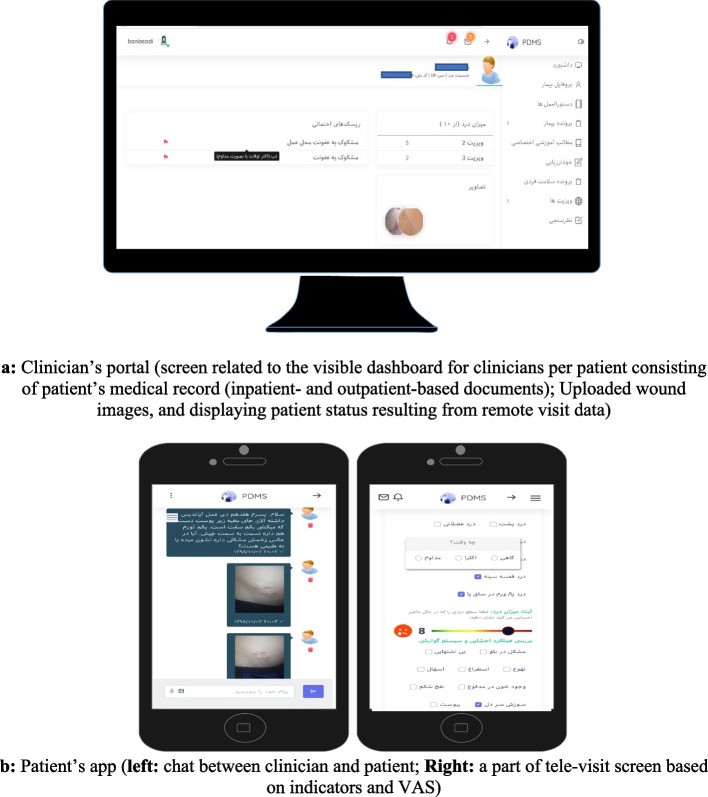
Sample screenshots from wound telemonitoring system. a: Clinician’s portal (screen related to the visible dashboard for clinicians per patient consisting of patient’s medical record (inpatient- and outpatient-based documents); Uploaded wound images, and displaying patient status resulting from remote visit data). b: Patient’s app (left: chat between clinician and patient; Right: a part of tele-visit screen based on indicators and VAS)

Sample screenshots from different parts of the developed platform are represented in the following figures (Fig. 4).

### Pilot test

The important results achieved from this phase were reported in the following sections.

#### Usage of the app

During the pilot test of the program, out of 15 registered patients, two men living in the city setting were recognized as inactive users. It was due to not logging in and not working with the application. Finally, 13 people completed the first and second remote visits. Likewise, 11 patients registered data up to the third visit, four people up to the fourth visit, and one person up to the fifth visit. In addition, two patients undergoing appendectomy requested and registered an emergency visit out of scheduled visits. Finally, patients were monitored and followed through the platform for 30 days after discharge. During telephone calls with patients who had not completed all 5 scheduled visits, we found that most of them had recovered and did not report any particular concerns.

5 discharged patients also raised their questions and concerns via chat service, and the questions were answered by the physician or nurse in the shortest possible time. Wound images were also received from four patients, and the quality of all six images was accepted by clinical specialists. It is noteworthy that only one patient reported a technical problem while working with the program, which was resolved. In general, the level of patients' participation in using the program was acceptable and satisfactory.

#### Practical interventions

Generally, interventions (automated or non-automated) provided to patients during the follow-up period included the following items:

- Sending automated messages and alerts to raise awareness
- Sending various reminders
- Sending recommendations messages from a doctor or nurse to high-risk patients (which could include returning to the surgery clinic or hospital)
- Providing counseling and answering patients' questions by clinicians
- If the clinician detected any anomaly finding on image review or in registered responses by patients, they called the patient for sending more documents or surgical wounds images.
- Matching the reported items by patients at possible risks with findings from telephone inquiries during the follow-up period.

According to findings, through this procedure, many problems and concerns of patients can be solved through nurse-centered interventions. In our study, these mainly included post-discharge unnecessary and routine concerns, nutritional challenges, an appropriate time to remove and dress sutures, bathing and traveling time, worries about the appearance of the wound, taking pain relievers, antibiotics, wound healing ointment, and anti-histamine for itching of the incision site.

#### Pilot usability evaluation

It is worth mentioning that in the present study, the evaluation activities were performed simultaneously or before the test phase. In general, all processes related to evaluation were presented in this section.

Based on the qualitative evaluation of the primary interface prototype by the experts’ panel, the reforms proposed by the potential users consist of the following items: changing some expressions, classifying the data elements, adding some useful links, shifting the icons in the user interface, using radio buttons vs drop-down menus or vice versa. Furthermore, clinicians emphasized the integration of the current program with the hospital information system (HIS) to prevent the re-entry of pre-recorded data. The feedback helped us to improve the design of the main version of the system.

Moreover, the qualitative evaluation of the initial version of the developed system by end users resulted in minor adjustments to the system but no major changes in features or database. For example, the patients suggested using easier ways to enter data and set up reminders such as drop-down lists, checkboxes, toggle switches, and the date and time pickers. In addition, in terms of visibility of system status, they emphasized color contrast for distinguishing active icons from inactive ones on the user interface.

We considered Nielsen's 10 heuristics in design processes. However, to ensure the maximum utilization of the factors derived from Nielsen's principles, the developed system was qualitatively evaluated by an expert panel. This stage involved five experts in the fields of medical informatics with a clinical base (*n* = 1), management and health information technology (*n* = 2), and one clinician with information technology skills. The average age and the average work experience of the participants (four males and one female) were 38.6 and 15 years, respectively. Results showed all ten heuristics were almost acceptable in the current system. However, some principles, such as aesthetic and minimalist design, needed to be addressed more in the updated version, especially in the admin panel and dashboard assigned to it.

Finally, the result of the usability quantitative evaluation by 10 patients and 10 healthcare providers is represented in Table 5. Subjects who met inclusion criteria in the previous phase subsequently entered the usability evaluation phase. The characteristics of the participants are presented in Table 4. Abdominal surgery procedures included appendectomy (*n* = 3), cholecystectomy (*n* = 3), gastrectomy (*n* = 1), diagnostic laparotomy (*n* = 2), and herniorrhaphy (*n* = 1).

**Table 5:** The system usability based on the patients' and healthcare providers' perspective

**Users**	**Age (means ± SD)**	**Gender: n (%)**	**Groups: n (%) (Physician or Nurse) /(Urban or Rural)**	**Indexes** (**Means ± SD**)		
				Ease of use and Satisfaction/ 7 Q	Arrangement and Classification of information/6 Q	Usefulness/ 7 Q
Healthcare providers	38.30 ± 4.94	M: 4 (40) F: 6 (60)	P: 4 (40) N: 6 (60)	4.19 ± 0.45	4.23 ± 0.52	4.49 ± 0.49
Patients	28.41 ± 3.1	M: 3 (30) F: 7 (70)	U: 4 (40) R: 6 (60)	4.13 ± 0.38	4.00 ± 0.49	4.20 ± 0.58
Total	33.35 ± 4.02	M: 7 (35) F: 13 (65)	-	4.16 ± 0.41	4.1 ± 0.50	4.34 ± 0.53

The average score related to the usefulness of the system was higher than the other two indicators, with an average score of 4.34 out of 5. This average score in the healthcare providers group is also higher than the patients' group (4.49 vs. 4.20).

According to the result of the pilot usability evaluation, high users’ satisfaction and willingness to use the program were achieved.

## Discussion

The telemedicine and mobile-based medical apps can be a valuable solutions in postoperative care management as they can well support patients and clinicians in improving care quality [40]. In this study, we developed an electronic system for post-discharge follow-up in abdominal surgery by focusing on SSIs monitoring. This system includes a web-based app for surgery patients (or family caregivers) and a web-based portal as remote monitoring and follow-up platform for healthcare providers. Results of the pilot test indicated applying this new technology potentially is feasible along with the understanding of the usefulness of the program by its users.

In line with our research, several studies have been performed to monitor postoperative wounds [5, 13, 19, 35, 40]. Thus, as the first step, we extracted and validated commonly used features in research-based mHealth apps for consideration in the current study. Accordingly, the key features and functions of the developed system included patient education in accordance with discharge instructions, reminders, notifications, capturing patient-centered clinical documents, virtual systematic tele-visits for regular assessment of self-reported symptoms, and uploaded wound images. According to evidence, uploading wound images, along with answering questions related to the signs and symptoms of the infection can be effective to help promptly identify infections. In studies by Gunter et al. [5] and Sood et al. [13], evidence showed that the smartphone-based app can facilitate the early detection of SSI and intervention in wound complications.

In addition to the above-mentioned features, based on end-user needs we also included more components and features in the design of the platform such as links to outpatient clinics and home nursing care services. In addition, user-preferred content was considered for the patient education module.

On the other hand, notifying clinicians, surveillance on remote visits via the fast-tracking control panel, and automatic monitoring with rule-based dashboards as well as teleconsultation are other functions of our system. We realized that visualization tools and rule-based dashboards could be very effective in the desire of clinicians to better interact with the system. Therefore, more efforts should be made to create creativity and apply intelligence-based analytics techniques in clinical dashboard design.

We regularly followed up the patients for 30 days after discharge through the developed platform, which was similar to the studies of Scheper et al. [40] Symer et al. [41] in terms of the monitoring period. In the current study, thirteen patients actively participated in the pilot test phase. In total, out of 5 scheduled tele-visits, 11 patients (85%) completed at least 2 visits during 30 days. Generally, the response rate to tele-visits decreased over time from the first to the last visit and this response pattern has been observed in other studies as well [2, 50]. In our study, most patients who completed subsequent visits still had at least one problem or concern about their recovery.

The findings of this study confirmed that patient-provider communication was facilitated. According to patients’ feedback, follow-up-oriented teleconsultations have helped them to feel more supported and more confident. Indeed, our platform and those like it allows patients to be in better real-time communication with providers via secure connections. These facilitated communications in precedingstudies have been identified as a priority by patients [5, 35].

Based on the results of the evaluation in the present study, the ease of use factor obtained a desirable and acceptable score. In most similar studies, patients and providers' satisfaction and a positive attitude towards the use of mobile-based follow-up apps were reported. In addition, easy-to-use apps were another reason for the desire to use them [5, 19, 35, 40]. Therefore, simple design along with approaches related to user-centered design should be followed with more emphasis on the design of any healthcare-related app. Importantly, understanding the importance of the usefulness of the program by its potential users is a valuable achievement in the current study. It helps to increase the desire for more effective use of the system. On the other hand, the treatment team and patients should receive adequate training to work with a new system that helps to overcome barriers to acceptability.

Despite the prospective potential for future use of telemedicine and mobile apps for postoperative follow-up, some important challenges remain. On network security and patient rights, since data from a web-based mobile app immediately is available on a hospital server, it requires ethical handling with security and privacy considerations [51]. In the meantime, common policies such as a multirole authority management setting for the care team's terminal, or password management in the patient’s terminal may help to protect data security and patient privacy [45].

In general, a growing number of recent studies have shown the utilization of home care mobile apps as a new modality of out-hospital care. This has been accompanied by potential benefits for monitoring recovery, providing postoperative follow-up support for early detection of complications, adherence to treatment and postoperative instructions, self-management, and subsequent reduction in readmissions [21, 43]. In addition, evidence has shown the potential of these digital solutions, particularly for people who live in rural or less privileged areas or had mobility difficulties [43, 52]. All these benefits should encourage Iran's health system for targeted investment and comprehensive planning to set up this type of post-discharge management system. This will most likely have positive outcomes and many advantages in the long run.

Overall, our study showed that a customized platform with this diversity of data collection and a wide range of functions could be developed successfully to establish the feasibility of using the system by its potential end-users. Furthermore, we provided primary evidence that surgery wound telemonitoring is a feasible solution and acceptable manner for patients and clinicians in our study setting. Therefore, priority planning is needed to make the program more operational.

Furthermore, we found many of the patients' concerns were solved by nurse-centered interventions alone; it highlighted the role of nurses as the main stakeholder of the system. Therefore, this clinical group should be more involved in the process of developing the system. Finally, the reported findings from our experience during development and pilot testing can help professionals who are looking to use this system and even similar systems in Iranian hospitals.

## Strength and limitations

To our best knowledge, the developed system was one of the first serious efforts to apply technology-based surgical wound telemonitoring in Iran that could be assumed as the strength of this research. Another strong point of our work was the close involvement of stakeholders in the phases of system design and development. Although this process was time-consuming, it played an important role in achieving more comprehensive documentation of system requirements, prioritizing the end-user needs and user-centered design policies.

This study has several limitations. A major limitation of the current study was the initial evaluation of the system usability with a small sample size of the population, which potentially limits its generalizability. The evaluation of the proposed system should be performed based on multiple aspects with the involvement of more experts and different groups of patients. More usability tests and next evaluations are needed. Accordingly, a robust evaluation of the system’s effectiveness is proposed before proceeding to larger-scale implementation. These will be useful for the achievement of valuable information to upgrade the system as well. Next, after implementation on a larger scale, it will be possible in future studies to investigate its impact on clinical outcomes based on measurable indicators.

## Conclusion

Applying a surgical wound telemonitoring system potentially is feasible in our study setting and any similar setting in Iran. The users had great satisfaction and willingness to use the system to diminish unnecessary exposure and in-person outpatient visits. Via this platform, patients directly and actively engage in their care, which can lead to a movement toward shared decision-making and patient-centered care. This technology was not intended to change the routine care standards. This new wound evaluator as part of routine postoperative care management can provide positive effects and outcomes. It is highlighted especially in the era of COVID-19 or any other similar situation in the future, because at this time more willingness for telehealth-based and virtual care services is seen.

## Supplementary Information

**Additional file 1.** 

**Additional file 2.** 

## Data Availability

All data generated or analyzed during this study are included in this published article.
