# Supplementary material for: Developing a mobile health application for wound telemonitoring: a pilot study on abdominal surgeries post-discharge care
Source: BMC Med Inform Decis Mak. 2023 Jun 2;23:103. doi: 10.1186/s12911-023-02199-z (PMC10237057; doi:10.1186/s12911-023-02199-z)
Supplement: Supplementary file 1 — Additional file 1. [file 12911_2023_2199_MOESM1_ESM.docx]

**Appendix I**

**Delphi Questionnaire for validation of system components**

Dear doctor/nurse

The present questionnaire has been developed after studying the literature and checking the forms available in the hospitals and expert opinions to determine the minimum information content of post-surgery follow-up system. It is worth mentioning that this project will be implemented as a pilot in patients undergoing open abdominal surgery with a priority and a focus on the prevention and identification of surgical site infections.

The questionnaire includes three sections (basic components, indexes for wound evaluation, and risk model). Additionally, at the end of each section, a free text box is considered for recording any new experts’ suggestions. The questionnaire items were scored based on a five-option Likert scale (from 1 to 5); the scores of 5 and 1 belong to the most important and least important items, respectively.

Please specify your valuable opinions regarding the "level of importance" of each item mentioned in the questionnaire.

Thanks

Best regards

**Characteristics of the Experts' Panel**

- **Age** ................. years / - **Gender:** female ⃝ male ⃝

- **Work experience:** less than 5 years ⃝ 5 to 10 years ⃝ 10 to 20 years ⃝ more than 20 years ⃝

- **Current position:**

Nurse⃝ Head nurse ⃝ Supervisor ⃝ Matron ⃝ Other................................... **Education level** ...........................

Intern ⃝ General practitioner ⃝ Resident ⃝ Specialist ⃝ Subspecialist ⃝ **Specialized field** ..............................

Other: .................................................

**Supplementary Table-S1:** The degree of importance of items and components of the system based on the expert's perspective

| **Parts of system** | **5-point Likert scale**  **Mobile App components** | **The degree of importance** | | | | |
| --- | --- | --- | --- | --- | --- | --- |
|  |  | Very unimportant (1) | Unimportant (2) | Somewhat important (3) | Important (4) | Very important (5) |
| **Patient Portal** | Surgery-related documents |  |  |  |  |  |
|  | Self-management service |  |  |  |  |  |
|  | Remote follow-up visits |  |  |  |  |  |
|  | Patient-provider communication service |  |  |  |  |  |
|  | Patient-patient communication service and sharing experience |  |  |  |  |  |
| Other suggested items for this section |  | | | | | |
|  | **5-point Likert scale**  **Web-based platform components** | Very unimportant (1) | Unimportant (2) | Somewhat important (3) | Important (4) | Very important (5) |
| **Care Team Portal** | Discharge Instructions and clinical data management |  |  |  |  |  |
|  | Educational content management |  |  |  |  |  |
|  | Smart monitoring and surveillance dashboard |  |  |  |  |  |
|  | Intervention management and telecommunication |  |  |  |  |  |
|  | Educational panel for clinicians |  |  |  |  |  |
|  | Static reporting |  |  |  |  |  |
| Other suggested items for this section |  | | | | | |

**Supplementary Table-S2:** The degree of importance of surgical wound evaluation indicators

| **Criteria** | **5-point Likert scale**  **Indexes** | Very unimportant (1) | Unimportant (2) | Somewhat important (3) | Important (4) | Very important (5) |
| --- | --- | --- | --- | --- | --- | --- |
| **Specific symptoms of wound infection** | Increased redness |  |  |  |  |  |
|  | Warmth |  |  |  |  |  |
|  | Tenderness |  |  |  |  |  |
|  | High swelling, bulging, stiffness, and inflammation |  |  |  |  |  |
|  | Wound drainage (Green, White, Yellow) |  |  |  |  |  |
|  | Foul smell from the wound |  |  |  |  |  |
|  | Wound dehiscence |  |  |  |  |  |
|  | High bleeding of wounds |  |  |  |  |  |
|  | Sudden and exaggerated pain at the surgical site (use of analgesic) |  |  |  |  |  |
|  | Presence of prominent red lines |  |  |  |  |  |
|  | Acne and white blisters around the wound |  |  |  |  |  |
|  | Irritation |  |  |  |  |  |
|  | Dryness |  |  |  |  |  |
|  | Darkening and thickening of the skin |  |  |  |  |  |
|  | Large and deep wounds |  |  |  |  |  |
| Other suggested items for this section |  | | | | | |
| **General Symptoms of infection** | Fever |  |  |  |  |  |
|  | Chills |  |  |  |  |  |
|  | unusual sweating |  |  |  |  |  |
|  | Dizziness |  |  |  |  |  |
|  | Weakness |  |  |  |  |  |
|  | Fatigue |  |  |  |  |  |
|  | Lethargy |  |  |  |  |  |
|  | Pains |  |  |  |  |  |
| Other suggested items for this section |  | | | | | |
| **Overall satisfaction with wound healing process** | Dissatisfaction, relative satisfaction, or complete satisfaction |  |  |  |  |  |
| **Surgical wound images** | Qualitative assessment of uploaded wound images |  |  |  |  |  |
| Other suggested items for this sections |  | | | | | |

**Supplementary Table-S3:** The degree of importance of rules related to the evaluation of Infection and SSI^*^

| Rules | | | | The degree of importance | | | | |
| --- | --- | --- | --- | --- | --- | --- | --- | --- |
| IF | | **THEN** | | Very unimportant | Unimportant | Somewhat important | Important | Very important |
| Symptom | **Occurrence /Frequency** | **Alert type: Flag** | **Risk type** | (1) | (2) | (3) | (4) | (5) |
| Fever/Chills | Yes/ [Most of the time or Always] | 🏱 (Red) | Infection |  |  |  |  |  |
| Wound drainage (Green, White, Yellow) | Yes/ [Evident at the self-assessment time] | 🏱 (Red) | SSI^*^ |  |  |  |  |  |
| High swelling, bulging, stiffness, and inflammation | Yes/ [Evident at the self-assessment time] | 🏱 (Red) | SSI |  |  |  |  |  |
| Wound dehiscence | Yes/ [Evident at the self-assessment time] | 🏱 (Red) | SSI |  |  |  |  |  |
| High bleeding of wounds | Yes/ [Evident at the self-assessment time] | 🏱 (Red) | SSI |  |  |  |  |  |
| Weakness and lethargy | Yes [Always] | 🏱 (Yellow) | Infection |  |  |  |  |  |
| Sudden and increasing pain at the surgical site | Yes/ [Most of the time] | 🏱 (Red) | SSI |  |  |  |  |  |
| Abdomen pain and around it | Yes/ [Most of the time or Always] | 🏱 (Yellow) | SSI |  |  |  |  |  |
| Wound drainage & Abdominal Pain | Yes &Yes / [Evident at the self-assessment time & Always] | 🏱 (Red) | SSI |  |  |  |  |  |
| Increased redness of the surgical incision or the skin around it & Warmth of the incision site | Yes &Yes / [Evident at the self-assessment time] | 🏱 (Red) | SSI |  |  |  |  |  |
| Increased redness of the surgical incision or the skin around it & Wound drainage | Yes &Yes / [Evident at the self-assessment time] | 🏱 (Red) | SSI |  |  |  |  |  |
| Increased redness of the surgical incision or the skin around it & Pain in the abdomen and around it | Yes &Yes / [Evident at the self-assessment time & Most of the time or Always ] | 🏱 (Red) | SSI |  |  |  |  |  |
| Weakness or lethargy & Fever | Yes &Yes / [Always & Sometimes] | 🏱 (Yellow) | Infection |  |  |  |  |  |
|  |  |  |  |  |  |  |  |  |
| Other suggested rules for this section |  | | | | | | | |

***SSI: Surgical Site Infection**
