# Supplementary material for: Developing a mobile health application for wound telemonitoring: a pilot study on abdominal surgeries post-discharge care
Source: BMC Med Inform Decis Mak. 2023 Jun 2;23:103. doi: 10.1186/s12911-023-02199-z (PMC10237057; doi:10.1186/s12911-023-02199-z)
Supplement: Supplementary file 2 — Additional file 2. [file 12911_2023_2199_MOESM2_ESM.docx]

**Appendix II**

**The mHealth App Usability Questionnaire (MAUQ)**

**Appendix II.A**

**MAUQ to study the usability of the surgical patients' follow-up application: Patient's point of view:**

This questionnaire is designed to collect your opinions about the usability of the application for monitoring and follow-up of surgery patients.

Your answers will only be available to the researchers of project. To better evaluating please after working with the application, make your decision in answering the questions quickly and don't think too much about it so that your real feeling is recorded. If you don't think an option can be evaluated, you do not answer it. Remember that this is your personal opinion, there is no right or wrong answer. The questionnaire items were scored based on a five-option Likert scale from 1(Strongly disagree) to 5 (Strongly agree). After reading each question carefully, choose and mark the desired option according to your agreement or disagreement.

Thank you for your cooperation and for giving us your time.

**Characteristics of patients**

- **Age** ................. years / - **Gender:** female ⃝ male ⃝

**Living place:** Urban ⃝ Rural ⃝

**Marital status:** Single ⃝ Married ⃝ Divorced or separated ⃝

**Occupation:** Student ⃝ Administrative Personnel ⃝ Farmer ⃝ Worker ⃝ Retired or Disabled ⃝ Other ⃝ Not employed ⃝

**Education:** High school ⃝ High school diploma ⃝ Associate degree ⃝ Bachelor’s degree and higher ⃝ Other ⃝

**Supplementary Table-S1:** Usability of the surgical patients' follow-up application based on patient's perspective

| **Categories** | **Items** | **Strongly disagree** | **Disagree** | **Neither agree nor disagree** | **Agree** | **Strongly agree** |
| --- | --- | --- | --- | --- | --- | --- |
| **Ease of use and satisfaction**  **(MAUQ_E)** | I1. The app was easy to use. |  |  |  |  |  |
|  | I2. It was easy for me to learn to use the app. |  |  |  |  |  |
|  | I3. I like the interface of the app. |  |  |  |  |  |
|  | I4. The information in the app was well organized, so I could easily find the information I needed. |  |  |  |  |  |
|  | I5. The amount of time involved in using this app has been fitting for me. |  |  |  |  |  |
|  | I6. I would use this app again. |  |  |  |  |  |
|  | I7. Overall, I am satisfied with this app. |  |  |  |  |  |
| **System information arrangement**  **(MAUQ_S)** | I8. Whenever I made a mistake using the app, I could recover easily and quickly. |  |  |  |  |  |
|  | I9. This mHealth app provided an acceptable way to receive health care services. |  |  |  |  |  |
|  | I10. The app adequately acknowledged and provided information to let me know the progress of my action. |  |  |  |  |  |
|  | I11. The navigation was consistent when moving between screens. |  |  |  |  |  |
|  | I12. The interface of the app allowed me to use all the functions (such as entering information, responding to reminders, viewing information) offered by the app. |  |  |  |  |  |
|  | I13. This app has all the functions and capabilities I expect it to have. |  |  |  |  |  |
| **Usefulness**  **(MAUQ_U)** | I14. The app would be useful for my health and well-being. |  |  |  |  |  |
|  | I15. The app improved my access to health care services. |  |  |  |  |  |
|  | I16. The app helped me manage my health effectively |  |  |  |  |  |
|  | I17. The app made it convenient for me to communicate with my health care provider. |  |  |  |  |  |
|  | I18. Using the app, I had many more opportunities to interact with my health care provider. |  |  |  |  |  |
|  | I19. I felt confident that any information I sent to my provider using the app would be received. |  |  |  |  |  |
|  | I20. I felt comfortable communicating with my health care provider using the app. |  |  |  |  |  |

**Appendix II.B**

**MAUQ to study the usability of the surgical patients' follow-up application: Healthcare provider's point of view**

This questionnaire is designed to collect your opinions about the usability of the interactive application for monitoring and follow-up of surgery patients.

Your answers will only be available to the researchers of project. Please, to better evaluate please after working with the application, make your decision in answering the questions quickly and don't think too much about it so that your real feeling is recorded. If you don't think an option can be evaluated, you do not answer it. Remember that this is your personal opinion, there is no right or wrong answer. The questionnaire items were scored based on a five-option Likert scale from 1(Strongly disagree) to 5 (Strongly agree). After reading each question carefully, choose and mark the desired option according to your agreement or disagreement.

Thank you for your cooperation and for giving us your time.

**Characteristics of healthcare provider**

- **Age** ................. years / - **Gender:** female ⃝ male ⃝

- **Work experience:** less than 5 years ⃝ 5 to 10 years ⃝ 10 to 20 years ⃝ more than 20 years ⃝

- **Current position:**

Nurse⃝ Head nurse ⃝ Supervisor ⃝ Matron ⃝ Other......................... **Education level:** ............................

Intern ⃝ General practitioner ⃝ Resident ⃝ Specialist ⃝ Subspecialist ⃝ **Specialized field**: ....................

**Experience working with the Hospital Information System (HIS):** Yes ⃝ ........ years, No ⃝

**Supplementary Table-S2:** Usability of the surgical patients' follow-up application based on healthcare providers' perspective

| **Categories** | **Items** | **Strongly disagree** | **Disagree** | **Neither agree nor disagree** | **Agree** | **Strongly agree** |
| --- | --- | --- | --- | --- | --- | --- |
| **Ease of use and satisfaction**  **(MAUQ_E)** | I1. The app was easy to use. |  |  |  |  |  |
|  | I2. It was easy for me to learn to use the app. |  |  |  |  |  |
|  | I3. I like the interface of the app. |  |  |  |  |  |
|  | I4. The information in the app was well organized, so I could easily find the information I needed. |  |  |  |  |  |
|  | I5. The amount of time involved in using this app has been fitting for me. |  |  |  |  |  |
|  | I6. I would use this app again. |  |  |  |  |  |
|  | I7. Overall, I am satisfied with this app. |  |  |  |  |  |
| **System information arrangement**  **(MAUQ_S)** | I8. Whenever I made a mistake using the app, I could recover easily and quickly. |  |  |  |  |  |
|  | I9. This app provided an acceptable way to *deliver* health care services. |  |  |  |  |  |
|  | I10. The app adequately acknowledged and provided information to let me know the progress of my action. |  |  |  |  |  |
|  | I11. The navigation was consistent when moving between screens. |  |  |  |  |  |
|  | I12. The interface of the app allowed me to use all the functions (such as entering information, responding to reminders, viewing information) offered by the app. |  |  |  |  |  |
|  | I13. This app has all the functions and capabilities I expect it to have. |  |  |  |  |  |
| **Usefulness**  **(MAUQ_U)** | I14. The app would be useful for my health *care practice*. |  |  |  |  |  |
|  | I15. The app improved my access to *delivering* health care services. |  |  |  |  |  |
|  | I16. The app helped me manage my *patients’* health effectively. |  |  |  |  |  |
|  | I17. The app made it convenient for me to communicate with my *patients***.** |  |  |  |  |  |
|  | I18. Using the app, I had many more opportunities to interact with my *patients*. |  |  |  |  |  |
|  | I19. I felt confident that any information I sent to my *patients* using the app will be received. |  |  |  |  |  |
|  | I20. I felt comfortable communicating with my *patients* using the app. |  |  |  |  |  |
